# Evaluation of cerebral perfusion heterogeneity by the electrical impedance tomography

**DOI:** 10.3389/fphys.2024.1476040

**Published:** 2024-11-13

**Authors:** Mingxu Zhu, Yu Wang, Junyao Li, Weice Wang, Guobin Gao, Zhenyu Ji, Benyuan Liu, Lei Wang, Weichen Li, Xuetao Shi

**Affiliations:** ^1^ Department of Biomedical Engineering, Air Force Medical University, Xi’an, China; ^2^ Shaanxi Provincial Key Laboratory of Bioelectromagnetic Detection and Intelligent Perception, Department of Biomedical Engineering, Air Force Medical University, Xi’an, China; ^3^ Institute of Medical Research, Northwestern Polytechnical University, Xi’an, China; ^4^ Faculty of Electrical and Control Engineering, Liaoning Technical University, Huludao, China; ^5^ Functional and Molecular Imaging Key Lab of Shaanxi Province, Department of Radiology, Tangdu Hospital, Air Force Medical University, Xi’an, China

**Keywords:** cerebral perfusion, electrical impedance tomography, inhomogeneity index, asymmetry index, heterogeneity

## Abstract

**Purpose:**

The purpose of this study was to evaluate the ability of global inhomogeneity index (GI) and left-right asymmetry index (AI) based on electrical impedance tomography (EIT) to be used in assessing cerebral perfusion heterogeneity. The diagnostic value of these two indices in identifying abnormalities in the degree of cerebral perfusion heterogeneity was also explored.

**Methods:**

In this study, Transcranial Doppler (TCD) was used as a control, and unilateral carotid artery was compressed to change the degree of heterogeneity of cerebral perfusion in 15 healthy volunteers. The control group consisted of an additional 15 volunteers without any intervention. EIT perfusion images were obtained by calculating the impedance difference between at the beginning and end of cerebral vasodilation. Subsequently, GI and AI were calculated based on the pixel values of intracranial regions.

**Results:**

The GI and AI values in the non-carotid artery compression (NCAC) group were significantly lower than those in the unilateral carotid artery compression (UCAC) group (P < 0.001), whereas there was no significant difference between the left carotid artery compression (LCAC) and right carotid artery compression (RCAC) groups. ROC analysis showed that the area under the curve (AUC), specificity and sensitivity of GI in distinguishing between NCAC and UCAC were 0.94, 0.90 and 0.87, respectively. The AUC, specificity and sensitivity of AI in distinguishing between NCAC and UCAC were 0.86, 0.87 and 0.73, respectively.

**Conclusion:**

The results demonstrated that the GI and AI effectively quantify the distribution of intracranial perfusion, demonstrating excellent validity and interindividual comparability, and the ability to detect abnormal cerebral perfusion heterogeneity.

## 1 Introduction

Cerebral perfusion heterogeneity, i.e., the phenomenon of uneven distribution of blood flow in different regions of the brain, is common in a variety of diseases, such as stroke, brain tumors, epilepsy, etc (Gelfand, Wintermark, and Josephson, 2010; Larsson et al., 2017). These disorders may result in abnormal blood flow in specific areas, including significant increases or decreases, which in turn may affect the normal functioning and metabolic activity of brain cells (Wegener et al., 2024). Lin et al. and T Mustonen et al. showed that heterogeneity of cerebral perfusion was associated with thromboembolic events. They confirmed the validity of cerebral perfusion heterogeneity as a marker to predict the occurrence of cerebrovascular accidents (Mustonen et al., 2008; Lin et al., 2021). Therefore, monitoring cerebral perfusion heterogeneity is essential for identifying brain diseases and evaluating the course of treatment, helping to improve the quality of life of patients and reduce the incidence of associated complications.

Clinical detection of perfusion heterogeneity usually relies on Computed Tomography (CT) (Copen, Lev, and Rapalino, 2016), Magnetic Resonance Imaging (MRI) (Le et al., 2024) and Positron Emission Tomography (PET) (Baron, 2022). They provide not only high-resolution images, but also functional information such as blood flow, metabolic activity and tissue perfusion. However, widely application of these techniques is limited in that they require contrast media and are expensive, making them difficult to use for continuous noninvasive bedside monitoring. In addition, while TCD can provide continuous, noninvasive monitoring of cerebral perfusion heterogeneity, its application is limited by the fact that it can only monitor the major blood vessels in the brain, and is unable to provide simultaneous perfusion of multiple brain regions (Lau et al., 2020). Therefore, the development of a whole-brain noninvasive, real-time, bedside cerebral perfusion heterogeneity monitoring technique is of great need.

Electrical impedance tomography (EIT) is a non-invasive functional imaging technique that measures boundary voltages at body surfaces to estimate the spatial distribution of the electrical properties (conductivity or resistivity) of tissue within a body (Bayford, 2006). This technology has shown potential for a wide range of applications in several medical fields including lung function imaging (Frerichs et al., 2017), brain function imaging (Aristovich et al., 2016) and abdominal organ imaging (Sadleir and Fox, 2001). Zhang et al. proposed the contrast-enhanced EIT technique that can reflect the cerebral perfusion status by the reconstructed image of the contrast agent, and found that unilateral internal carotid artery occlusion has a significant variability in the left and right hemisphere cerebral impedance values (Zhang et al., 2022). By performing contrast-enhanced EIT imaging on a rabbit model of focal cerebral infarction, Zhang et al. demonstrated that EIT can detect the location and area of different cerebral infarct lesions by monitoring cerebral perfusion (Zhang et al., 2023). The dynamic cerebral perfusion indices extracted from EIT reconstructed images by Yan et al. were able to characterize the differences in cerebral perfusion status at different intracranial pressure levels, proposing a new approach to intracranial pressure monitoring (Yan et al., 2024). The above findings show that diseases can cause changes in the distribution of perfusion impedance and fully demonstrate the unique advantages of EIT technology in cerebral perfusion monitoring. EIT not only has a high temporal resolution that provides long-term continuous monitoring of intracranial perfusion, but is also a completely noninvasive and harmless monitoring technique. However, there is a lack of indices for assessing cerebral perfusion heterogeneity that can be used for interindividual comparisons because EIT images from different individuals show only relative impedance values and cannot be directly compared. Therefore, there is a great need for quantitative indices to measure the degree of cerebral perfusion heterogeneity.

In this study, the heterogeneity of cerebral perfusion was altered by compression of the carotid artery using TCD as a control. We extracted indices from EIT perfusion images aimed at characterizing the distribution of cerebral perfusion and evaluated their interindividual comparability. The ability of these indices in detecting abnormally elevated cerebral perfusion heterogeneity was further explored.

## 2 Materials and methods

### 2.1 Study protocol

**FIGURE 1:**
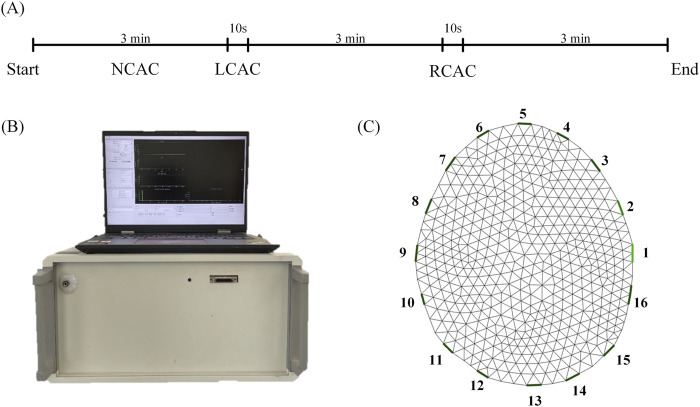
The experimental process. **(A)** Experiment time. **(B)** EIT system. **(C)** A finite element reconstructed model. NCAC: Non-carotid artery compression; LCAC: Left carotid artery compression; RCAC: Right carotid artery compression.

The study was conducted in the laboratory of the Department of Biomedical Engineering, Fourth Military Medical University and was approved by the Research Ethics Committee of the Fourth Military Medical University (FMMU-E-III-001 (1–7)). We included 30 healthy volunteers, all of whom were fully aware of the methodology of this study and signed a consent form. Prior to the start of the experiment, 16 Ag/AgCl electrodes were connected to the subject’s head to collect impedance information. Electrode No. 1 was located 2 cm above the left ear, electrode No. 5 was located at the top of the forehead, electrode No. 9 was located 2 cm above the right ear, and electrode No. 13 was located at the back of the head in the occipital region. The electrode bands were located on the same profile and were equally spaced (Figure 1C). A medical elastic bandage is tightly wrapped around the electrode to temporarily block the effects of scalp blood flow while assisting in securing the electrode. Meanwhile, TCD probes (Coggin Industries, Nanjing, China) were fixed in the subjects’ bilateral temporal windows to monitor the blood flow velocity in the bilateral middle cerebral arteries.

Thirty volunteers were divided into two groups. 15 volunteers were included in the control group and their data were collected for 3 min without compression of the carotid artery (NCAC). 15 volunteers changed the distribution of perfusion by compressing the carotid artery. The experimental procedure was as follows: first, at the beginning of the experiment, we collected 3 min of baseline data. Then, a 10-s compression of the left carotid artery was performed (LCAC). At the end of the left carotid artery compression, subjects were placed on a 3-min rest to allow their physiology to return to near normal levels. At the end of the rest, we performed the same compression procedure on the right carotid artery (RCAC) (Figure 1A).

### 2.2 EIT system

The EIT signals were collected using a new jointly developed EIT system (Figure 1B, UTRON Technology Co., Ltd., Hangzhou, China), which is based on a high-speed, high-precision system developed by our team (Shi et al., 2018; Li et al., 2019). The system has an operating frequency range of 10–250 kHz, an output current range of 10–1,250 μA, and a signal-to-noise ratio greater than 90 dB. During the experiments, data collection speed was set to 40 frames per second, and the excitation frequency was set to 50 kHz.

### 2.3 EIT image reconstruction

The finite element model was dissected based on CT images of the human brain, which contained 881 triangular surface elements (Figure 1C). In order to improve the imaging quality, the conductivity of the scalp (0.584 S/m), skull (0.0084 S/m), and brain parenchyma (0.2849 S/m) was included as *a priori* information in the EIT reconstruction process (Ouypornkochagorn et al., 2022). Reconstruction of conductivity distributions is an underdetermined and ill-posed problem that usually requires simplifying assumptions or regularization based on *a priori* knowledge. This study used a maximum *a posteriori* probability method of linearized image reconstruction using the noise variance of the measured data and the covariance of the conductivity distribution (Adler and Guardo, 1996; Adler and Lionheart, 2006), as shown in Equation 1:

$$∆\sigma ={\left(H^{T}WH+\lambda R\right)}^{-1}H^{T}W∆V$$ (1)

where ∆σ is the change in conductivity distribution between the current frame and the reference frame, ∆V is the change in voltage between the current frame and the reference frame, λ is the regularization parameter, W is the measurement error weighting matrix, H is the sensitivity matrix, R is the regularized matrix.

**FIGURE 2:**
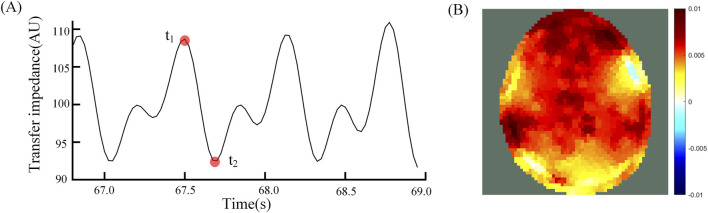
**(A)** Average transmission impedance of 192 channels, t1 is the reference time and t2 is the foreground time; **(B)** Differential EIT images at time t1 and t2.

The analysis of the subsequent results of this study was performed based on EIT perfusion images. Perfusion images were generated by reconstructing the image at the end of vasodilation (Figure 2A t2) using the time of the start of cerebral vasodilation (Figure 2A t1) as the reference frame. Figure 2A t1 was the start of the vasodilatory. When the vasodilation occurs and blood enters the cranial cavity, a decrease in overall impedance is observed due to the significant difference in resistivity of blood compared to brain tissue. The time that the impedance value dropped to the lowest point (Figure 2A t2) indicated that vasodilation had peaked and blood perfusion into the cranial cavity was maximized. The EIT perfusion images captured at this time (Figure 2B) were able to reflect the state of blood perfusion in the cranial cavity.

### 2.4 Calculation of indices

For each perfusion cycle, a 64 × 64 perfusion image is generated. Each pixel of the perfusion image represents the difference in impedance at the beginning and end of vasodilation. The entire region of the brain parenchyma is defined as the perfusion region. Each pixel value in this region is counted and its median is calculated. This yields the sum of the absolute difference between all pixel values and this median is a way to characterize the variability of blood flow distribution across the perfusion region, a metric known as the global inhomogeneity index (Zhao et al., 2009). To ensure that the cerebral perfusion inhomogeneity index is comparable across individuals, as well as to better generalize its application, the index needs to be normalized, i.e., divided by the sum of impedance values within the perfusion region. They can be calculated using the following Equation 2:

$$GI=\sum_{x,y\in ROI}\left|{∆\sigma }_{xy}-Median\left({∆\sigma }_{ROI}\right)\right|÷\sum_{x,y\in ROI}{∆\sigma }_{xy}$$ (2)

where ∆σ denotes the pixel value in the EIT perfusion image; ${∆\sigma }_{xy}$ is the pixel value in the perfusion region; ${∆\sigma }_{ROI}$ is all the pixel values in the perfusion region.

The pixel values of the perfusion regions of the perfusion images were normalized, i.e., the mean was subtracted from the original values and divided by the standard deviation for normalized comparisons. The images were then divided into regions of the left and right hemispheres along the midline of the brain. For each row of the EIT image, we calculated the mean values of the pixels on the left and right sides separately, which in turn yielded the differences between the left and right means on the corresponding row. The sum obtained by adding up the absolute values of all the differences reflects the total difference in the degree of perfusion between the left and right hemispheres, which can be used as a measure of brain symmetry. Define the brain midline $x=x_{0}$ and divide the image into left L and right R. AI was calculated by Equation 3:

$$AI=\sum_{y_{i}}\left|\frac{1}{N_{L,i}}\sum_{\left(x,y_{i}\right)\in L}{∆\sigma }_{norm}\left(x,y_{i}\right)-\frac{1}{N_{R,i}}\sum_{\left(x,y_{i}\right)\in R}{∆\sigma }_{norm}\left(x,y_{i}\right)\right|$$ (3)

where ${∆\sigma }_{norm}$ is the normalized ∆σ ; $\left.L=\{{\left(x,y\right)\;|\;x>x}_{0}\right\}$ is the left region; $\left.R=\{{\left(x,y\right)\;|\;x<x}_{0}\right\}$ is the right region; $N_{L,i}$ and $N_{R,i}$ are the number of pixels in the ith row in the left and the right regions, respectively, $y_{i}$ denotes the ith row.

### 2.5 TCD control

In the present experiment, blood flow velocities in the left and right brain were monitored synchronously using the TCD technique with the aim of assessing the degree of cerebral perfusion heterogeneity. This was done to help validate the effect of carotid artery compression on cerebral perfusion heterogeneity. For in-depth analysis, the ratio of peak systolic flow rate (V_s_) to diastolic flow rate (V_d_), the so-called S/D ratio, was calculated. This ratio is an important indicator of vascular elasticity and vascular resistance. To quantify the degree of heterogeneity in cerebral perfusion, the following normalization Equation 4 was used:

$$R=\frac{\left|N_{S/D}-C_{S/D}\right|}{N_{S/D}}$$ (4)

where $N_{S/D}$ is the S/D ratio of the non−compressed side of the cerebral hemisphere; $C_{S/D}$ is the S/D ratio of the cerebral hemisphere on the side of compression. The degree of heterogeneity in cerebral perfusion was calculated in the NCAC state using the equation $R=\frac{\left|R_{S/D}-L_{S/D}\right|}{L_{S/D}}$ . Larger R-values indicate a higher degree of heterogeneity in cerebral perfusion.

### 2.6 Statistical analysis

Statistical analysis was performed using SPSS27 (SPSS Inc., Chicago, IL, United States). The level of significance for statistical analysis was 0.05. The Shapiro-Wilk test was used to determine whether the data conformed to normal distribution. The Levene test was used to assess the Chi-squared goodness of variances between different groups.

Independent samples t-tests were used to compare the mean differences in GI and AI between NCAC and LCAC, and NCAC and RCAC, respectively. Paired t-tests were used to compare LCAC and RCAC. In addition, we constructed receiver operating characteristic curve (ROC) to assess the efficacy of two indices, GI and AI, in distinguishing NCAC from unilateral carotid artery compression (UCAC). This assessment is based on three main metrics: area under the curve (AUC), sensitivity and specificity.

## 3 Results

**FIGURE 3:**
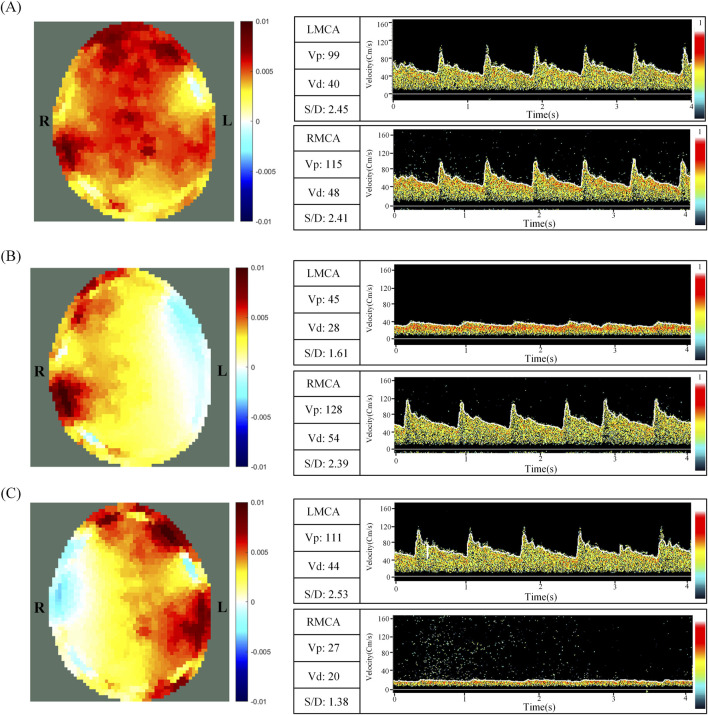
EIT perfusion images and TCD information for NCAC **(A)**, LCAC **(B)** and RCAC **(C)** states. L and R represent the left and right parts of the brain, respectively. NCAC: Non-carotid artery compression; LCAC: Left carotid artery compression; RCAC: Right carotid artery compression; LMCA: Left middle cerebral artery; RCMA: Right middle cerebral artery; V_p_: Peak systolic blood flow velocity; V_d_: Diastolic velocity; S/D: The ratio of peak systolic flow rate (V_p_) to diastolic flow rate (V_d_).

Figure 3 shows the EIT perfusion images and TCD information for NCAC, LCAC and RCAC states. A similar trend was clearly observed in the EIT images and TCD information of all the volunteers. The EIT perfusion image of NCAC state shows a more homogeneous distribution of blood flow in the intracranial region. This can also be seen in the subtle difference in blood flow velocities between the right and left brain measured by the TCD in Figure 3B. However, in Figures 3B, C, the blood flow distribution between the right and left intracranial regions appeared significantly different. Specifically, when the left carotid artery was compressed (as shown in Figure 3B), there was a significant reduction in the red area in the left intracranial region relative to the right side, suggesting a reduction in blood flow on that side. This was also verified by the fact that the cerebral blood flow velocity measured by TCD was significantly lower on the left side than on the right side at this time. Comparatively, when the right carotid artery was compressed (as shown in Figure 3C), the red area in the right intracranial region was reduced, indicating decreased blood flow on that side. Cerebral blood flow velocities were also smaller on the right side than on the left.

In the NCAC group, the TCD index R was 0.02 ± 0.02, indicating a low degree of flow heterogeneity in the perfused region. In contrast, the LCAC had an R value of 0.42 ± 0.11, whereas the RCAC had an R value of 0.49 ± 0.15. These higher values indicate a significant increase in the degree of heterogeneity of the perfusion distribution under carotid artery compression.

Tables 1, 2 demonstrate the GI and AI values for each volunteer in the NCAC, LCAC, and RCAC states. To ensure the accuracy of the comparison, we calculated the mean and standard deviation of five perfusion cycles in each state separately. The results showed that the GI and AI in the NCAC state had high stability, which was reflected in their low standard deviations. However, the standard deviation of these two indices increased significantly in the LCAC and RCAC states, which may be due to differences in applied pressure as well as interindividual differences in compensatory function.

**TABLE 1:** GI of 15 volunteers of the control group and 15 volunteers during carotid compression.

Group	GI values (mean ± SD)				
NCAC	0.55 ± 0.02	0.35 ± 0.03	0.37 ± 0.01	0.49 ± 0.01	0.46 ± 0.02
	0.50 ± 0.01	0.37 ± 0.03	0.52 ± 0.10	0.68 ± 0.12	0.37 ± 0.07
	0.41 ± 0.05	0.51 ± 0.02	0.34 ± 0.02	0.33 ± 0.02	0.49 ± 0.02
	Average mean 0.45; Average SD 0.04				
LCAC	0.66 ± 0.04	1.43 ± 0.06	0.62 ± 0.05	0.54 ± 0.07	0.73 ± 0.14
	0.64 ± 0.10	0.95 ± 0.14	0.99 ± 0.14	1.12 ± 0.16	0.52 ± 0.07
	1.03 ± 0.05	0.64 ± 0.04	0.90 ± 0.13	0.66 ± 0.20	0.70 ± 0.18
	Average mean 0.81; Average SD 0.10				
RCAC	0.69 ± 0.08	0.76 ± 0.04	0.65 ± 0.02	0.90 ± 0.33	0.67 ± 0.07
	0.57 ± 0.12	1.01 ± 0.12	0.89 ± 0.07	0.96 ± 0.08	0.45 ± 0.04
	0.59 ± 0.01	0.69 ± 0.07	0.50 ± 0.05	0.53 ± 0.20	1.32 ± 0.60
	Average mean 0.75; Average SD 0.13				

**TABLE 2:** AI of 15 volunteers of the control group and 15 volunteers during carotid compression.

Group	AI values (mean ± SD)				
NCAC	3.42 ± 0.27	5.57 ± 1.2	4.36 ± 0.33	4.68 ± 0.68	3.27 ± 0.15
	5.04 ± 0.15	5.82 ± 0.59	7.88 ± 2.5	4.52 ± 0.64	4.73 ± 1.59
	6.01 ± 1.02	4.80 ± 1.58	4.48 ± 0.49	5.39 ± 1.12	4.18 ± 0.85
	Average mean 4.94; Average SD 0.88				
LCAC	13.47 ± 0.47	10.78 ± 1.47	6.86 ± 0.95	5.60 ± 0.79	5.66 ± 0.70
	7.53 ± 0.65	5.81 ± 0.30	9.42 ± 0.48	5.45 ± 0.51	4.61 ± 1.16
	10.64 ± 2.36	11.56 ± 1.68	9.54 ± 2.33	7.61 ± 3.04	5.02 ± 0.91
	Average mean 7.97; Average SD 1.19				
RCAC	11.56 ± 1.11	9.94 ± 0.59	6.46 ± 1.04	3.34 ± 0.39	5.85 ± 1.38
	8.63 ± 0.72	10.77 ± 0.80	8.30 ± 0.61	7.08 ± 0.63	5.34 ± 0.94
	10.36 ± 2.09	8.02 ± 0.63	6.30 ± 1.06	5.80 ± 2.45	5.4 ± 1.28
	Average mean 7.54; Average SD 1.05				

**FIGURE 4:**
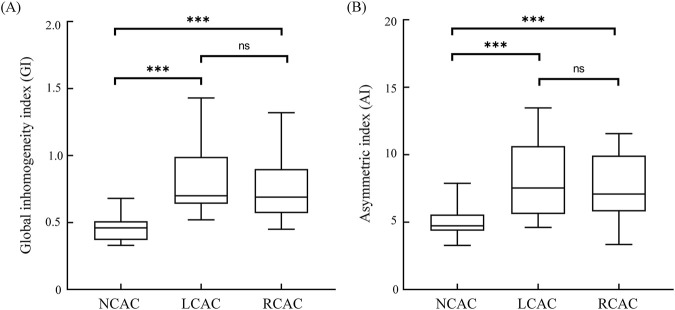
GI **(A)** and AI **(B)** for NCAC, LCAC and RCAC. Boxes represent quartiles, i.e., the top edge of the box represents the third quartile (Q3) and the bottom edge the first quartile (Q1). The whiskers of the box-and-line plots extend from the ends of the boxes, and the ends of the whiskers represent the maximum and minimum values of the data. ns: no significant difference; * p < 0.05; *** p < 0.001. NCAC: non-carotid artery compression; LCAC: left carotid artery compression; RCAC: right carotid artery compression.

Figure 4 demonstrates the results of the comparison of the two indices GI and AI for NCAC, LCAC and RCAC. The results showed that the GI of NCAC, was significantly different from the GI of LCAC and RCAC, which was statistically significant (p < 0.001), whereas no significant difference was found when comparing the GI of LCAC and RCAC. For AI, its performance in different states was similar to GI. Specifically, the AI values of NCAC were significantly different from those of LCAC and RCAC (P < 0.001), while no significant difference was found between the AI values of LCAC and RCAC.

**FIGURE 5:**
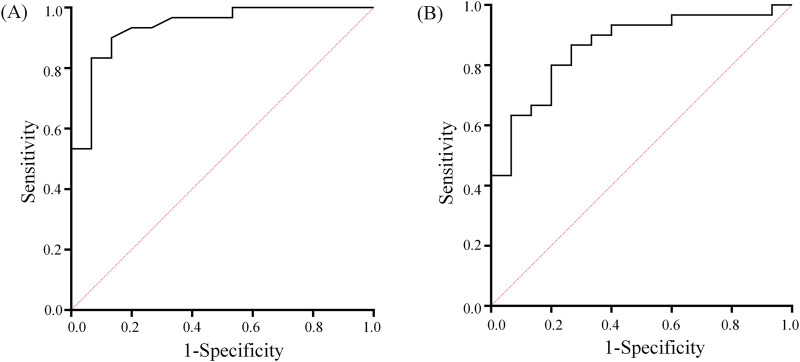
GI **(A)** and AI **(B)** ROC curves used to distinguish between NCAC and UCAC. The diagonal line indicates the random probability (0.5).

ROC analysis showed (Figure 5) that the critical value of GI in distinguishing NCAC from UCAC was 0.525. Its AUC, specificity and sensitivity were 0.94, 0.90 and 0.87, respectively. The critical value of AI in distinguishing NCAC from UCAC was 5.395. Its AUC, specificity and sensitivity were 0.86, 0.87 and 0.73, respectively.

## 4 Discussion

Many brain diseases, such as brain tumors, strokes, and cerebrovascular lesions, have the potential to affect the degree of cerebral perfusion heterogeneity. Assessing and characterizing the degree of cerebral perfusion heterogeneity is critical in the diagnosis and treatment of these diseases. Existing cerebral perfusion monitoring techniques have multiple limitations that make it difficult to effectively realize real-time noninvasive monitoring of the degree of cerebral perfusion heterogeneity. EIT has outstanding advantages in terms of high temporal resolution and noninvasiveness, which offer the possibility of solving the limitations of existing techniques. However, there is a lack of indices that can assess cerebral perfusion heterogeneity and are suitable for interindividual comparisons. In this study, EIT imaging was performed on the NCAC, LCAC, and RCAC status of the volunteers, i.e., perfusion images were generated between the beginning and end of vasodilatation. Using these perfusion images, we extracted two indices to characterize the state of cerebral perfusion and compared these two indices between NCAC, LCAC, and RCAC. In addition, we evaluated the ability of these indices to distinguish between NCAC and UCAC through the ROC.

Carotid artery compression significant effects cerebral blood flow, and it is a validated clinical procedure often used by physicians to assess cerebral autoregulation (CA) (Smielewski et al., 1996). Carotid artery compression is an excellent way to verify the sensitivity of EIT to changes in cerebral hemodynamics. Shi and Ouypornkochagorn et al. reported 2D and 3D EIT imaging during carotid artery compression in human volunteers, respectively, and verified the sensitivity of EIT to cerebral hemodynamics (Shi et al., 2018; Ouypornkochagorn et al., 2022). In clinical practice, carotid artery compression is commonly used in conjunction with TCD to assess cerebral autoregulation (CA). Given that the temporal window is very sensitive to blood flow velocities in the region of the middle cerebral artery, TCD becomes an ideal control tool for validating the validity of indices (GI and AI) extracted from EIT perfusion images that reflect cerebral perfusion heterogeneity. In this study, we used TCD as a control means to assess the cerebral perfusion heterogeneity of NCAC, LCAC and RCAC. By analyzing the TCD, we extracted the index R, which reflects the degree of cerebral perfusion heterogeneity, and thus verified the effect of carotid artery compression on the aspect of cerebral perfusion heterogeneity.

Figure 3 clearly demonstrates the differences between NCAC, LCAC, and RCAC on EIT perfusion imaging; these images visually reveal the significant effects of carotid artery compression on cerebral perfusion heterogeneity and confirm the ability of the EIT technique to accurately capture these changes. Analysis of the indices showed that the two key indices were significantly different between NCAC and LCAC and RCAC (Figure 3), a result that is consistent with the heterogeneity of cerebral perfusion embodied in TCD. It is worth noting that NCAC has a smaller interquartile spacing between the two indices compared to UCAC, which may stem from inter-individual physiologic differences as well as differences in the degree of carotid artery compression. This variability can likewise be captured by the standard deviation of the TCD index. By analyzing the ROC curves of GI and AI (Figure 4), we identified their potential value in detecting excessive degrees of cerebral perfusion heterogeneity. The above results not only demonstrate the stability and inter-individual comparability of GI and AI, but also show their potential application in detecting heterogeneous abnormalities of brain perfusion under disease.

Substantial progress has been made in the field of cerebral perfusion research with EIT. Currently, the technique is divided into two main monitoring methods: contrast-enhanced EIT and dynamic cerebral perfusion EIT. A study by Zhang et al. utilized contrast-enhanced EIT with contrast media in an animal model of carotid artery occlusion and revealed significant differences in perfusion between the unoccluded and occluded sides of the carotid artery (Zhang et al., 2022). Similarly, Zhang et al. successfully identified significant differences in perfusion between infarcted regions and normal brain tissue using contrast-enhanced EIT technique (Zhang et al., 2023). These findings not only highlight the potential application of EIT in brain perfusion research, but also indirectly demonstrate the ability of EIT to monitor brain perfusion heterogeneity. However, although these studies identified perfusion differences between lesions and healthy tissues on EIT images, they failed to propose an index that could accurately quantify the distribution of cerebral perfusion impedance.

Yan et al. monitored cerebral perfusion changes during elevated intracranial pressure using the dynamic cerebral perfusion EIT technique and extracted several monitoring indices from reconstructed images that could accurately characterize the differences in cerebral perfusion under changes in intracranial pressure (Yan et al., 2024). However, these indices are affected by multiple factors such as blood pressure, which limits their comparability between individuals and makes them more suitable for comparing changes in different physiological states in a single individual. The two indices extracted from the perfusion images in this study contain information about each pixel in the EIT image and can accurately characterize the degree of heterogeneity in brain perfusion distribution. Although the dynamic cerebral perfusion EIT technique was used in this study, both indices can also be applied in contrast-enhanced EIT.

This study has some limitations. (1) In this study, the distribution of cerebral perfusion was altered by compression of the carotid artery, which in turn validated the extracted indices. However, this method does not directly reflect the true cerebral perfusion distribution in disease states. For a more comprehensive understanding of brain perfusion heterogeneity under different pathological conditions, future studies should be expanded to include in-depth studies of stroke, brain tumors, and other diseases that affect brain perfusion distribution. (2) In examining cerebral perfusion heterogeneity in this study, we focused mainly on the inhomogeneity of global perfusion distribution and the left-right asymmetry of cerebral perfusion. However, the distribution patterns of cerebral perfusion are likely to be more complex and diverse, and other under-explored distributional features exist. Therefore, future studies should broaden their horizons to explore more dimensions of cerebral perfusion distribution and develop a more detailed and comprehensive parameter system based on them, with a view to more accurately characterizing and understanding the complexity of cerebral perfusion.

## 5 Conclusion

This study verifies that the GI and AI indices for assessing the degree of cerebral perfusion heterogeneity are highly reliable and stable for inter-individual comparisons. In addition, these indices can accurately characterize the distribution of cerebral perfusion in different states and have a certain detection value, which is expected to be an important tool for clinical assessment and treatment. At the same time, GI and AI metrics may also play an important role in other situations affecting cerebral perfusion, such as evaluating the effectiveness of thrombolysis, assessment of collateral circulation, and cerebral function studies, among other areas.

## Data Availability

The raw data supporting the conclusions of this article will be made available by the authors, without undue reservation.
